# *Rabaptin-5* and *Rabex-5* are neoplastic tumour suppressor genes that interact to modulate Rab5 dynamics in *Drosophila melanogaster*^☆^

**DOI:** 10.1016/j.ydbio.2013.09.029

**Published:** 2014-01-01

**Authors:** Chloe Thomas, David Strutt

**Affiliations:** MRC Centre for Developmental and Biomedical Genetics, Department of Biomedical Science, University of Sheffield, Western Bank, Sheffield S10 2TN, UK

**Keywords:** Rab5, Rabaptin5, Rabex5, *Drosophila*, Neoplastic tumour suppressor, Endocytosis

## Abstract

Endocytosis plays an important role in the regulation of tumour growth and metastasis. In *Drosophila*, a number of endocytic neoplastic tumour suppressor genes have been identified that when mutated cause epithelial disruption and over-proliferation. Here we characterise the *Drosophila* homologue of the Rab5 effector Rabaptin-5, and show that it is a novel neoplastic tumour suppressor. Its ability to bind Rab5 and modulate early endosomal dynamics is conserved in *Drosophila*, as is its interaction with the Rab5 GEF Rabex5, for which we also demonstrate neoplastic tumour suppressor characteristics. Surprisingly, we do not observe disruption of apico-basal polarity in *Rabaptin-5* and *Rabex-5* mutant tissues; instead the tumour phenotype is associated with upregulation of Jun N-terminal Kinase (JNK) and Janus Kinase (JAK)/Signal Transducer and Activator of Transcription (STAT) signalling.

## Highlights

• *Drosophila Rabaptin-5* and Rabex-5 are endocytic neoplastic tumour suppressor genes.
• The Rab5 effector function of Rabaptin-5 is highly conserved in *Drosophila*.
• Rabaptin-5 interacts with Rabex-5 to modulate early endosomal dynamics *in vivo*.
• Tumour phenotypes are associated with upregulation of JNK and JAK/STAT signalling.

## Introduction

The endocytic pathway influences many developmental processes including signalling, tissue morphogenesis and cell polarity. Although the endocytic process has been described in great detail in the mammalian cell culture, *in vivo* analysis of its specific developmental roles has been complicated by the essential and constitutive requirement for endocytosis in most cell types, and there remains much to be understood.

In *Drosophila*, many endocytic components have been found to function as neoplastic tumour suppressor genes (reviewed in Vaccari and Bilder (2009)). Loss of function mutations in these genes cause epithelial tissues to over-proliferate, differentiation to be lost and normal tissue architecture including apico-basal polarity, to be disrupted (Herz et al., 2009, 2006; Lu and Bilder, 2005; Menut et al., 2007; Moberg et al., 2005; Morrison et al., 2008; Rodahl et al., 2009; Thompson et al., 2005; Vaccari and Bilder, 2005; Vaccari et al., 2009).

Endocytic genes can be grouped into two categories with slightly different tumour phenotypes, according to whether the gene acts early or late in the endocytic pathway. Endocytic tumour suppressor genes such as *Rab5*, the syntaxin *Avalanche* (*Avl*) and the Rab5 effector *Rabenosyn-5* (*Rbsn-5*), act at the level of early endosome fusion (Gorvel et al., 1991; Lu and Bilder, 2005; Morrison et al., 2008; Nielsen et al., 2000). Despite the tumour phenotype of tissue wholly mutant for these genes, clones of mutant cells in an otherwise wild-type background do not overgrow and instead tend to become out-competed by wild-type cells and eliminated from the tissue (Lu and Bilder, 2005). Work in other tumour suppressor mutants in which the phenomenon was first observed, indicates that this probably occurs through a JNK-dependent apoptotic response, which after clone elimination allows tissues to resume their wild-type pattern of growth and development (Agrawal et al., 1995; Brumby and Richardson, 2003; Igaki et al., 2006, 2009; Ohsawa et al., 2011; Uhlirova et al., 2005). However, a second group of tumour suppressor genes, which act later in the endocytic pathway, exhibit a non-autonomous tumour phenotype whereby mutant clones have the ability to promote growth in adjacent wild-type tissue (Herz et al., 2006, 2009; Moberg et al., 2005; Thompson et al., 2005; Vaccari and Bilder, 2005; Vaccari et al., 2009; reviewed in Herz and Bergmann (2009)). These encode some of the components of the ESCRT (endosomal sorting complexes required for transport) complex, which functions in the formation of multivesicular bodies (MVBs, reviewed in Jouvenet (2012)). The difference between these two types of endocytic tumour seems to be due to their differential effects on Notch (N) signalling, caused by ectopic accumulation of N protein at different points of the endocytic pathway, depending upon where the endocytic block occurs (Herz et al., 2006, 2009; Lu and Bilder, 2005; Moberg et al., 2005; Morrison et al., 2008; Thompson et al., 2005; Vaccari and Bilder, 2005; Vaccari et al., 2008). In general, whilst lack of N internalisation in early endocytic mutants does not appear to promote signalling (Lu and Bilder, 2005; Morrison et al., 2008; Vaccari et al., 2008), blockage of entry into MVBs in ESCRT mutants causes N signalling to be ectopically activated, resulting in transcription of the secreted JAK/STAT ligand Unpaired (Upd), which acts as a transforming factor to promote growth in adjacent wild-type tissue (Herz et al., 2006, 2009; Moberg et al., 2005; Thompson et al., 2005; Vaccari and Bilder, 2005; Vaccari et al., 2008).

Neoplastic tumour phenotypes were first observed in mutants for *lethal giant larvae* (*lgl*), *discs large* (*dlg*) and *scribble* (*scrib*), components of the baso-lateral septate junction complex that is required to regulate apico-basal polarity of epithelial cells (Agrawal et al., 1995; Bilder et al., 2000; Bilder and Perrimon, 2000; Bryant and Schubiger, 1971; Gateff, 1978; Gateff and Schneiderman, 1967; Woods and Bryant, 1989; reviewed in Ellenbroek et al. (2012); Enomoto and Igaki, 2011). Various experiments indicate that over-expression of apical proteins such as Crumbs (Crb) or atypical protein kinase C (aPKC) can expand the apical domain, resulting in loss of apico-basal polarity and subsequent induction of neoplasia (Bilder and Perrimon, 2000; Leong et al., 2009; Wodarz et al., 1995). This has been proposed as a mechanism for tumourigenesis in the endocytic mutants, as Crb has been shown to accumulate abnormally in endocytic tumours (Lu and Bilder, 2005; Moberg et al., 2005; reviewed in Vaccari and Bilder (2009)). However, it is clear that compromising endocytosis will lead to abnormal trafficking of components of many developmental pathways and hence the aetiology of neoplastic tumour formation is likely to be complex.

Although at least 14 endocytic tumour suppressor genes have already been identified in *Drosophila*, screens have not been saturating and it is likely that many remain undiscovered. Here we describe a previously uncharacterised neoplastic tumour suppressor gene: the *Drosophila* homologue of *Rabaptin-5*, which we initially found in a screen to identify novel genes involved in planar polarity (Helen Strutt, Vickie Thomas-MacArthur, CT and DS, unpublished data). Rabaptin-5 is a Rab5 effector protein that has been shown to physically interact with activated Rab5 and promote homotypic early endosome fusion in the mammalian tissue culture (Horiuchi et al., 1997; McBride et al., 1999; Stenmark et al., 1995; Vitale et al., 1998). The latter function of Rabaptin-5 is mediated through formation of a complex with the Rab5 guanine nucleotide exchange factor (GEF) Rabex-5, which acts to increase nucleotide exchange on Rab5, enhancing its activity and hence its ability to recruit other effectors involved in the endosomal fusion process (Horiuchi et al., 1997; Lippe et al., 2001). In addition to Rabex-5, a large number of other binding partners for Rabaptin-5 have been characterised to date, and it appears that it can act divalently to bridge different trafficking pathways. For example, through interacting with both Rab5 and Rab4 it mediates the transfer of cargo from early endosomes back to the plasma membrane through the fast recycling pathway (Daro et al., 1996; van der Sluijs et al., 1992; Vitale et al., 1998). Unlike most of the endocytic tumour suppressor genes so far identified, *Rabaptin-5* has been implicated in human cancers, (Christoforides et al., 2012; Magnusson et al., 2001; Wang et al., 2009; Xiao et al., 1997), and thus the *Drosophila* mutant may represent a valuable model for the study of human tumourigenesis.

We find that several functions of Rabaptin-5 are conserved in *Drosophila*, including its GTP-dependent binding of Rab5, its ability to modulate endosomal dynamics and its interaction with Rabex-5. Our work suggests that *Drosophila Rabex-5* is also a neoplastic tumour suppressor gene and that the tumour phenotype of both mutants is associated with ectopic activation of JNK and JAK/STAT pathways.

## Materials and methods

### *Drosophila* stocks and genetics

The following *Drosophila* stocks were used; From Bloomington stock centre (described in FlyBase): *MS1096-GAL4*, *459.2-GAL4*, *ptc-GAL4*, *pnr-GAL4*, *UAS-Dcr2*, *cnbwsp*, *P{ry*^*+*^*Δ(2–3)}*, *Ubx-FLP*, *FRT42D P{arm-lacZ}*, *P{arm-lacZ} FRT80B, P{EPgy2}CG4030EY*^*21320*^ (a P-element insertion in *Rbpn5* 3′UTR), *Df(2R)F36* (a deficiency uncovering *Rbpn5*) and *Df(3L)BSC250* (a deficiency uncovering *Rabex-5*). *P{GD11194}v26367* and *P{GD14133}v46329* are RNAi lines (VDRC). *10xSTAT-GFP* (Bach et al., 2007), *Gbe-Su(H)-lacZ* (Furriols and Bray, 2001) and *puc*^*E69*^ (Martín-Blanco et al., 1998) are reporter lines (gifts from Martin Zeidler). *ex-lacZ* is a reporter line (a gift from Barry Thompson and Boedigheimer and Laughon, 1993). *stat92E*^*F*^ is a hypomorph (Baksa et al., 2002). *Rabex-5*^*ex42*^ is a hypomorph (a gift from Cathie Pfleger, Yan et al., 2010). Transgenes used were *UAS-Rabex-5:Myc* and *UAS-Rabex-5*^*DPYT*^*:Myc* (gifts from Cathie Pfleger and Yan et al., 2010), *UAS-YFP-Rab5*^*S43N*^ (Bloomington and Zhang et al., 2007). *UAS-Rbpn-5* was constructed by insertion of the *Rbpn-5* coding region from the cDNA clone LD23155 (BDGP) into pUASt (Brand and Perrimon, 1993). *pWIZ-Rbpn-5* is an RNAi line with no predicted off-targets and no homology to existing VDRC RNAi lines made by double inverted cloning of a 312 bp fragment of *Rbpn-5* into the pWIZ vector (Lee and Carthew, 2003) using the following primers:

LeftRNAiCG4030+XbaI:

GCATCTAGACACACCTAATGGTGGCG

RightRNAiCG4030+XbaI:

GCTCTAGAAAGCTGCTGTTCAGGGAG

Transgenics were generated by BestGene.

### RNAi screen

An *in vivo* RNAi screen was carried out using approximately 10,000 lines from Vienna Drosophila Research Centres (VDRC) and National Institute of Genetics (NIG) (Helen Strutt, Vickie Thomas-MacArthur, CT and DS, unpublished data). In brief, genes to be screened were pre-selected by FlyBase gene ontology, Interpro predicted protein domains and likely expression in the wing as predicted by microarray analysis (Ren et al., 2005). The *MS1096-GAL4* driver was chosen for initial screening due to its strong and relatively specific expression pattern in the *Drosophila* wing (Capdevila and Guerrero, 1994), with the *459.2-GAL4* driver used for secondary screening (both Bloomington). Screening was carried out at 29 °C and 25 °C. Adult wings were mounted in GMM mountant and scored for planar polarity defects.

### Generation of the *Rbpn-5* mutant

Male P-element mediated meiotic recombination was carried out as previously described (Preston et al., 1996), in flies carrying both the transposase *P*{*ry*^*+*^*Δ*(*2–3*)} and the *P{EPgy2}CG4030EY*^*21320*^ element, using *cn* and *bw* flanking markers for directional selection of recombination events. A small deletion, named *CG4030Del1*, was generated encompassing *CG4030*, *CG4038* and *CG34396* (sequence location 2R:16,976,092…16,989,307) and was found to be lethal if homozygous or transheterozygous with the larger deficiency *Df(2R)F36*.

In order to rescue the entire *CG4030Del1* deletion, the 33 kb *attB-P[acman]CH322-86L04* construct was obtained from BACPAC Resources and injected by Genetivision into the *P2(3L)68A4* site via a *Φ*C31 transposase-mediated transgenesis. Rescued flies were viable and appeared wild-type. Recombineering was used to generate a mutant form of *attB-P[acman]CH322-86L04* lacking the *CG4030* protein coding region, using methods based on those described by the Bellen lab (Venken et al., 2006 and protocols at www.pacmanfly.org/protocols.html). The following oligos were used to amplify the loxP cassette from *PL452N-EGFP*:

RightCG4030arm+LoxPR: **TGCATGGCTCGCCTTTGAATTAAACAACTCAATTCATTACTTTGATATCAA**CTAGTGGATCCCCTCGAGGGAC

LeftCG4030arm+LoxPF: **TCGGTATTATTCAAATATGGTAACACCAAGAGCAGTGTTGAAAATCGCTCTCGG**CATGGACGAGCTGTAC

The PCR fragment was recombineered into *attB-P[acman]CH322-86L04* and the *loxP* cassette was then flipped out to create *attB-P[acman]CH322-86L04-ΔCG4030*. The construct was verified by sequencing and injected by Genetivision into the *P2(3L)68A4* site via the *Φ*C31 transposase-mediated transgenesis. Transgenic flies were used to create the following genotype for analysis of the *Rbpn-5* mutant phenotype: *w*; *FRT42 CG4030Del1*; *attB-P[acman]CH322-86L04-ΔCG4030/SM6a:TM6b*

### Antibodies and immunohistochemistry

The following primary antibodies were used for immunolabelling: 1:50 rabbit anti-Rab5 (a gift from Marcos Gonzalez-Gaitan) (Wucherpfennig et al., 2003), 1:4000 rabbit anti-EGFP (Molecular Probes), 1:4000 rabbit anti-ß-Gal (Cappel), 1:1000 mouse anti-Flamingo (DSHB) (Usui et al., 1999), 1:300 rabbit anti-Frizzled (Bastock and Strutt, 2007), 1:1000 rat anti-Strabismus (Strutt and Strutt, 2008), 1:25 rat anti-Prickle (Strutt et al., 2013), 1:200 mouse anti-Armadillo N27A1 (DSHB) (Peifer et al., 1994), 1:20 rat anti-E-Cadherin (DSHB) (Oda et al., 1994), 1:250 guinea pig anti-Bazooka (a gift from Jennifer Zallen, and Wodarz et al., 1999), 1:100 mouse anti-Discs large 4F3 (DSHB) (Parnas et al., 2001), 1:500 rabbit anti-nPKC-zeta (cross reacts with *Drosophila* aPKC, Santa Cruz), 1:5 mouse anti-Crumbs Cq4 (DSHB) (Tepass et al., 1990), 1:200 rat anti-Elav 9F8A9 (DSHB), 1:50 mouse anti-Mmp1 catalytic domain 5H7B11 (DSHB) (Page-McCaw et al., 2003), 1:20 mouse anti-Notch (DSHB, a gift from Sarah Bray, and Fehon et al., 1991), 1:25 mouse anti-Myc 9E10 (Sheffield BioServe), 1:100 rabbit anti-Active JNK (Promega) and 1:1000 rabbit anti-Histone H3 (phospho S10) (Abcam).

To make anti-Rbpn-5, the first 750 bp of the *Rbpn-5* coding region was cloned into the *pGEX-6P-1* vector and expressed in *E.coli*. Soluble GST-Rpbn-5 antigen was purified on a Gluthathione Sepharose 4B column (GE Healthcare) and the GST tag was removed using PreScission Protease (Carl Smythe). 0.5 μg antigen was injected into each of three rats and serum from final bleeds was collected and used at 1:500 for immunohistochemistry. Secondary antibodies used were anti-rabbit, -rat, -mouse and -guinea pig Cy2, Alexa568 and Cy5 from Jackson and Molecular Probes.

For pupal wing dissection, white prepupae were collected and aged at 29 °C. Early pupal wings were dissected at 4.5 h after puparium formation (APF), when protein localisation is dynamic and cells are dividing, rearranging and changing shape, whereas late pupal wings were dissected at 26 h APF, a time point just before trichome formation when localisation of core planar polarity proteins is at its most asymmetric (Aigouy et al., 2010; Classen et al., 2005; Strutt and Strutt, 2009). Imaginal discs were taken from wandering 3rd instar larvae. Immunolabellings of imaginal discs and pupal wings were carried out as previously described (Strutt, 2001).

### GST pulldown assays

The coding regions of *Drosophila Rab4*, *Rab5*, *Rab5Q88L*, *Rab5N43S*, *Rab7*, *Rab8*, *Rab11*, *Rab23* and *Rabex5* were individually cloned into the *pGEX-6P-1* vector. GST-Rab proteins were expressed in *E.coli*, extracted from pelleted cells and bound to Glutathione Sepharose 4B beads (GE Healthcare) for 30  min at 4 °C in a buffer containing 50 mM Tris, pH 7.5, 50 mM NaCl, 5 mM MgCl_2_, and protease inhibitors. Beads were then incubated with GTP or GDP if required, for 90 min at room temperature in loading buffer containing 20 mM Hepes, 100 mM NaCl, 0.5 mM MgCl_2_, 2 mM EDTA, 0.05% CHAPS, 1 mM DTT and protease inhibitors.

*EGFP-Rbpn-5* was cloned into the *pAc5.1* vector (Invitrogen). The *Rbpn-5* fragments *Rbpn-5N* (amino acids 1–345) and *Rbpn-5C* (amino acids 340–642) were made by removal of the unwanted half of the gene from *pAc5.1-EGFP-Rbpn5* by restriction digestion, making use of the unique XhoI site halfway through the *Rbpn-5* coding region. For *Rbpn-5N* a linker was added containing a STOP codon. Gene fragments were EGFP-tagged and inserted into *pAc5.1*. Vectors were transfected into *Drosophila* S2 cells. Cell lysate was extracted 48 h after transfection and added to the freshly prepared Gluthathione Sepharose 4B beads. Beads were incubated for 2 h at 4 °C buffer, then washed in buffer containing 250 mM NaCl to remove non-specifically bound proteins. Beads were loaded directly onto 10% acrylamide gels after denaturing at 95 °C for 3  min and Western blots were carried out using 1:4000 rabbit anti-EGFP (Abcam) or 1:5000 rat anti-Rbpn-5. Secondary antibodies used were anti-rat and -rabbit HRP (Dako) and signal was detected using Supersignal West Dura (Thermo Scientific).

### Microscopy, image presentation and statistical analysis of puncta data

Fluorescent images were collected on an Olympus FV1000 confocal. Bright-field adult wing and notum photographs were taken using the ProgResC14 camera system from Jenoptik on a Leica DMR upright microscope. Images were processed in ImageJ and figures constructed using Adobe Photoshop.

For Rab5 puncta analysis, a Z stack of 0.15 μm slices at 60× magnification with 3× zoom (0.069 μm per pixel) was obtained and slices were maximally projected in ImageJ as follows. Apical was defined as the three most apical in focus slices (roughly 0.5 μm thickness), whereas subapical was defined as the next 0.5 μm (slices 4–6). To get a value for total Rab5 (Fig. 4G), the top 10 slices were projected. Projected images were thresholded in ImageJ using the Triangle function and particles were analysed for their number, average size and total area in identically sized wild-type and mutant areas of the wing. *T*-tests were used to calculate statistical significance. Mutant values were normalised against wild-type values within each wing to make graphs. Data was compiled and statistically analysed using the Microsoft Excel.

## Results

### A screen for genes involved in planar polarity identifies the *drosophila* homologue of *Rabaptin-5*

**Fig. S1:**
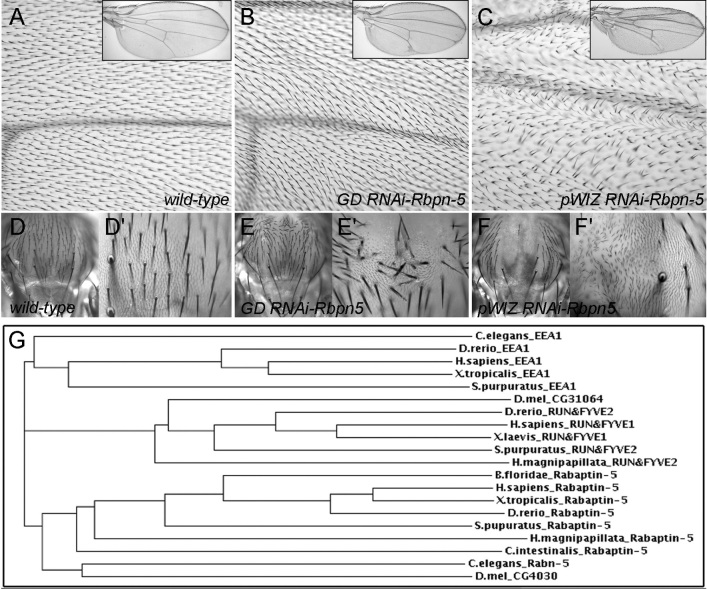
RNAi-Rbpn-5 causes planar polarity defects in wings and nota. (A) Magnification of a wild-type wing showing distally oriented trichomes. The L4 vein crosses the centre of the picture and the posterior cross vein is to the left. Inset shows entire wing. (B) Magnification of a wing expressing *GD26367 RNAi-Rbpn-5* with the *MS1096-Gal4* driver and *UAS-Dcr2*, showing posteriorly sloping trichomes. The L4 vein crosses the centre of the picture and the posterior cross vein is to the left. Inset shows entire wing with mild veining defects at L5 and a missing anterior cross vein. (C) Magnification of a wing expressing *pWIZ RNAi-Rbpn-5* with the *MS1096-Gal4* driver and *UAS-Dcr2*, showing swirling and duplicated trichomes. Some parts of the wing are slightly out of focus indicating wing unevenness. The L5 vein crosses the centre of the picture with L4 and the posterior cross vein at the top. Inset shows entire wing with veining defects at L5 and L2. All wings (A–C) are oriented with anterior upwards and distal to the right. (D) A wild-type notum showing the posterior orientation of macrochaetae, microchaetae and trichomes. D′ is a magnification of the central part of the notum. (E) A notum expressing *GD26367 RNAi-Rbpn-5* with the *pnr-Gal4* driver. The localisation of central microchaetae is disrupted and they are misoriented. The planar polarity of trichomes is also disrupted (E′). (F) A notum expressing *pWIZ RNAi-Rbpn-5* with the *pnr-Gal4* driver. Central microchaetae are lost and trichomes swirl and are often duplicated (F′). All nota (D-F) are oriented with anterior upwards. (G) A ClustalW alignment of Rabaptin-5, EEA1 and RUN&FYVE whole protein sequences from different organisms shows that *Drosophila* CG4030 clusters with Rabaptin-5 proteins.

**Fig. 1:**
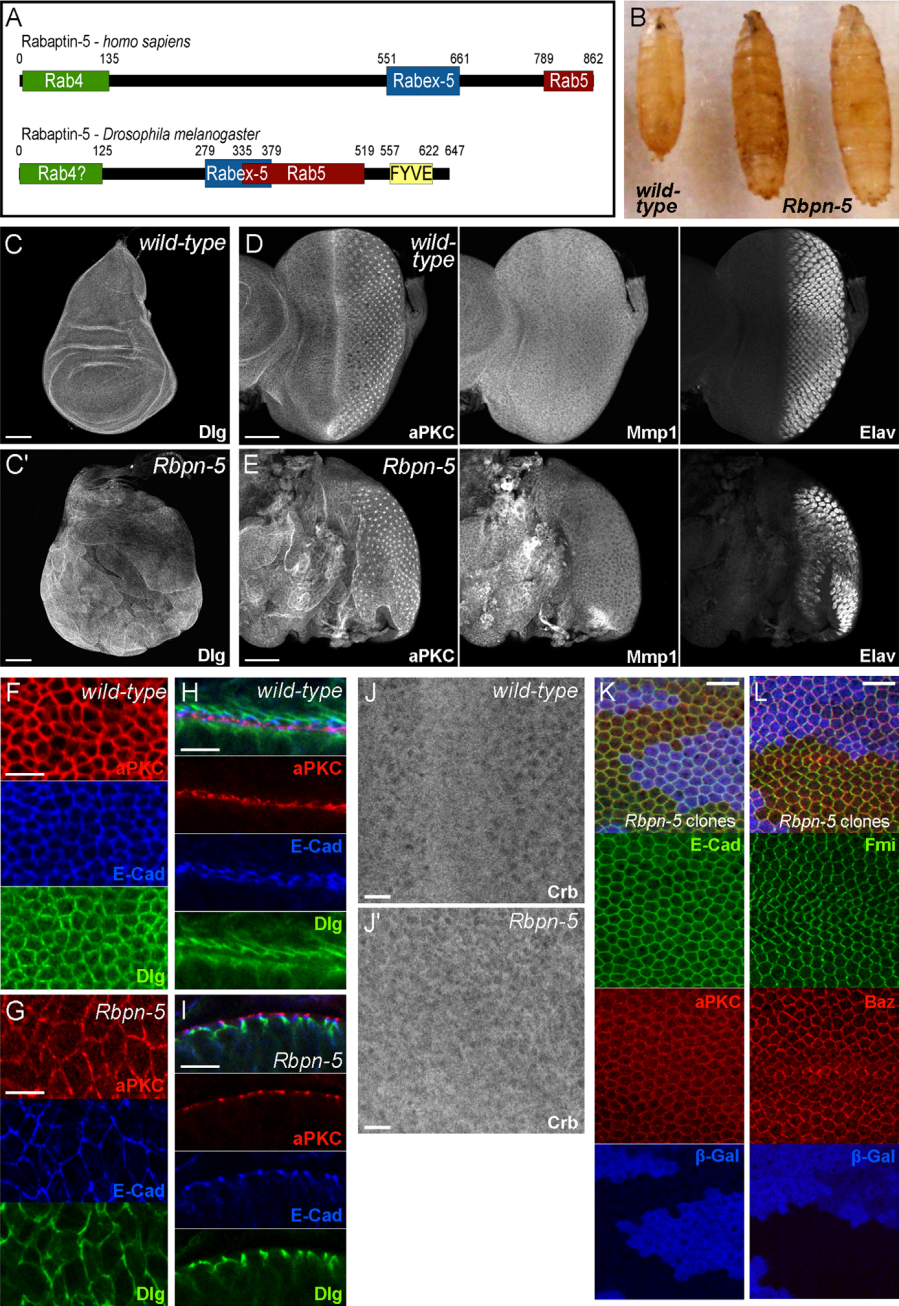
The *Drosophila* homologue of *Rabaptin-5* is a novel endocytic neoplastic tumour suppressor gene. (A) A cartoon of the human (isoform A) and *Drosophila* Rabaptin-5 proteins indicating functional domains determined experimentally for human Rabaptin-5 (Mattera et al., 2006; Vitale et al., 1998) and predicted by sequence homology for *Drosophila* Rbpn-5 (NCBI Conserved Domains). In *Drosophila* Rabaptin-5, the Rabex-5 and Rab4 binding domains are hypothetical and determined by BLAST of the equivalent domains in the human Rabaptin-5—BLAST scores are low (<40) for both. Rab5 binding domain (red), Rabex-5 binding domain (blue), Rab4 binding domain (green), FYVE domain (yellow). (B) Wild-type and *Rbpn-5* homozygous mutant pupae. The *Rbpn-5* mutant larvae grow considerably larger during extended larval stages, and die shortly after pupation. (**C**) Wild-type (C) and *Rbpn-5* homozygous mutant (C′) wing discs stained for Dlg. Scale bars are 100 μm long in both. *Rbpn-5* mutant tissue proliferates excessively to create a highly folded and disrupted epithelium. (D-E) Wild-type (D) and *Rbpn-5* homozygous mutant (E) eye discs stained for aPKC, Mmp1 and Elav. Differentiation is abnormal and Mmp1 levels are increased in the *Rbpn-5* mutant. Anterior is to the left. Scale bar is 50 μm long in both. (F-I) Wing discs stained with aPKC in red (apical), E-Cad in blue (junctional) and Dlg in green (baso-lateral) in wild-type (F,H) or *Rbpn-5* mutants (G,I). Although cells have a larger apical surface in *Rbpn-5* mutants than in wild-type (F-G), apico-basal polarity markers localise correctly at cell edges and show clear separation in a lateral view (H-I). In the wild-type (H), two apical surfaces are apposed in a fold of the wing disc, in the mutant (I), only a single apical surface is seen in an abnormal fold. Scale bars are 5 μm long in F–I. (J) Wildtype (J) and *Rbpn-5* homozygous mutant (J′) eye discs stained for Crb. Laser power and gain was kept at the same level for both images. Scale bars are 10 μm long. (K-L) Localisation of markers is normal in *Rbpn-5* mutant clones visualised in wings at 28 h APF. E-Cad is green and aPKC is red in K, Fmi is green and Baz is red in L. Clones are marked by absence of β-Gal staining (blue). Scale bars are 10 μm long in K and L.

We performed an *in vivo* RNAi screen in the *Drosophila* wing to look for novel genes involved in planar polarity (Helen Strutt, Vickie Thomas-MacArthur, CT and DS, unpublished data). An RNAi line targeting *CG4030* showed a phenotype of mild wing trichome swirling, together with mild veining defects (VDRC line GD26367, Fig. S1B). *CG4030* is predicted to encode a Rab5 binding protein with homology to Rabaptin-5 (Fig. 1A). Blast alignment indicates that CG4030 is likely to be the *Drosophila* Rabaptin-5 homologue (abbreviated here to Rbpn-5); protein homology is particularly high in the C-terminal region, which contains the putative Rab5 binding domain (NCBI Blast alignment score of 91.7), and is less conserved in the N-terminal region (NCBI Blast alignment score of 39.7). Unlike vertebrate Rabaptin-5 proteins, *Drosophila* Rbpn-5 has a FYVE (Fab1,YOTB/ZK632.12,Vac1 and EEA1) domain at its extreme C-terminus. FYVE domains target proteins to endosomal membranes through binding to phosphatidylinositol 3-phosphate (PI3P), a lipid that is enriched on endosomal membranes (Burd and Emr, 1998; Stenmark et al., 1996). The Rbpn-5 FYVE domain appears to be conserved in many invertebrates, indicating that it may have been lost at some point in the vertebrate lineage (data not shown). Interestingly, another Rab5-interacting, FYVE domain-containing protein, EEA1, is not found in arthropods, although is present in other invertebrates such as nematodes. However, sequence analysis suggests that *Drosophila* Rbpn-5 is only indirectly related to mammalian EEA1 through its FYVE domain and hence they are not likely to be orthologous (Fig. S1G).

To confirm the RNAi phenotype and check that the planar polarity phenotype was not due to an off-target effect, we made a separate *pWIZ-Rbpn-5* RNAi line targeting an independent sequence within the *Rbpn-5* gene with no predicted off-targets. This produced a phenotype of wing trichome swirling with frequent multiple trichomes produced per cell, in addition to mild wing veining defects and wing unevenness (Fig. S1C). We also analysed the effect of RNAi expression in the notum using the *pannier-GAL4* (*pnr-GAL4*) driver. *GD26367 RNAi-Rbpn-5* expression disrupts the polarity of microchaetae and trichomes in the notum, whereas pWIZ *RNAi-Rbpn-5* expression causes loss of microchaetae in addition to trichome polarity and spacing defects (Fig. S1E-F). These pleiotropic results suggest that Rbpn-5 acts in multiple processes.

### *Rbpn-5* is a neoplastic tumour suppressor gene

In order to analyse the function of Rbpn-5 in more detail, we removed gene activity entirely using a small deletion of the *Rbpn-5* gene region combined with p[acman] mediated rescue of flanking genes (see Section Materials and methods). *Rbpn-5* mutants have an extended larval stage (approximately doubled compared with wild-type at 25 °C) in which they grow significantly larger than wild-type larvae, before dying shortly after pupation (Fig. 1B). This ‘giant larvae’ phenotype is reminiscent of that seen in neoplastic tumour suppressor mutants, a subgroup of which correspond to mutations in genes encoding endocytic proteins including Rab5 itself and another Rab5 binding protein, Rbsn-5 (Lu and Bilder, 2005; Morrison et al., 2008).

To test whether *Rbpn-5* might be a novel neoplastic tumour suppressor gene, we examined wing and eye imaginal discs from *Rbpn-5* giant larvae. Discs are variable in size and many are no larger than wild-type discs, however, tissue structure is disrupted and the epithelium is highly overgrown and folded in on itself (Fig. 1C′,E). In addition, cell size appears to be variable, and many cells are extremely enlarged compared to wild-type (Fig. 1F-G). Neoplastic tumours are characterised by their absence of differentiation, disrupted apico-basal polarity and their upregulation of Matrix metalloprotease 1 (Mmp1), a target of JNK signalling involved in extracellular matrix remodelling, which is a marker for tumour metastasis (Beaucher et al., 2007; Page-McCaw et al., 2003; Srivastava et al., 2007; Uhlirova and Bohmann, 2006). Staining of eye discs with the neuronal marker Elav indicated that ommatidial differentiation does occur to some extent, but is not properly spatially regulated within the disc and differentiated cells appear abnormal in size and shape (Fig. 1E). Strong upregulation of Mmp1 levels was also seen, particularly in non-differentiated regions of the disc, indicating that tumours are likely to possess metastatic ability (Fig. 1E).

**Fig. S2:**
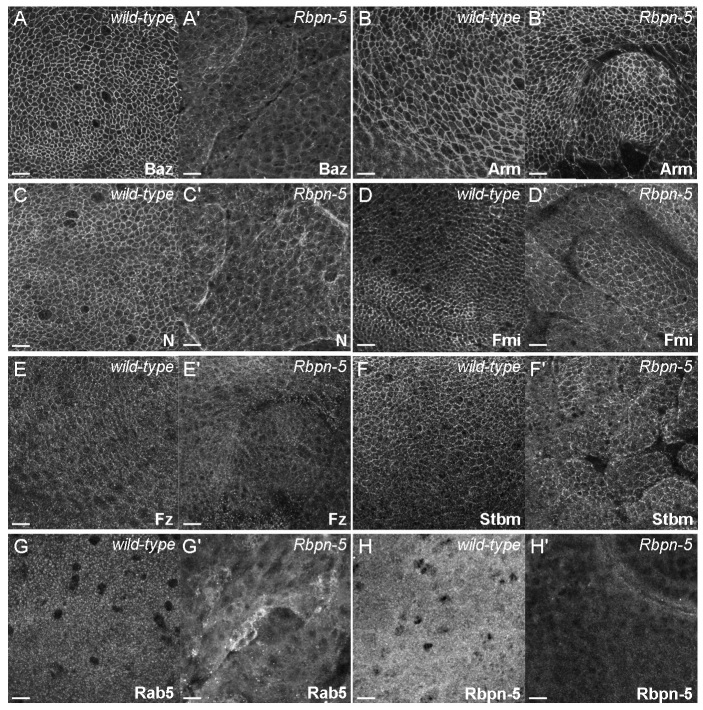
Localisation of markers in wild-type and *Rbpn-5* mutant wing discs. Wild-type (A-H) and Rbpn-5 mutant (A′–H′) discs. (A) Bazooka (Baz) localises to junctions in *Rbpn-5* mutants, although levels might be slightly lower than in wild-type wings. (B) Armadillo (Arm) localises normally to junctions in *Rbpn-5* mutant wings. (C) Notch (N) levels are normal and N localises to junctions in *Rbpn-5* mutant wings. (D–F) Core planar polarity proteins Flamingo (Fmi, D), Frizzled (Fz, E) and Strabismus (Stbm, F) localise to junctions at similar levels in wild-type and *Rbpn-5* mutants, although asymmetry is poor in mutant discs. (G) Rab5 localises to apical puncta in wild-type discs, but shows an abnormal clumped distribution in *Rbpn-5* mutant discs. (H) Levels of Rbpn-5 are strongly reduced in Rbpn-5 mutant discs. Residual staining may be due to remaining maternal protein or be non-specific. Scale bars in all panels are 10 μm long.

We examined the localisation of a large number of markers for apico-basal polarity, planar polarity and adherens junctions (Figs. 1F–J, S2A–F and not shown), all of which show a largely wild-type localisation pattern despite the disruption to tissue organisation. Unlike as described for other neoplastic tumour suppressors (Lu and Bilder, 2005; Menut et al., 2007; Moberg et al., 2005; Morrison et al., 2008; Rodahl et al., 2009; Vaccari and Bilder, 2005), it appears that apico-basal polarity is conserved in *Rbpn-5* mutants, as we saw clear separation of markers for apical (aPKC) and baso-lateral (Dlg) membrane domains (Fig. 1H-I). Loss of apico-basal polarity in other endocytic neoplastic tumour suppressor mutants has been proposed to occur through an expansion of the apical domain caused by a failure to endocytose the apical determinant Crb (Lu and Bilder, 2005; Moberg et al., 2005; reviewed in Vaccari and Bilder, 2009). To test whether localisation of Crb was affected in *Rbpn-5* mutants, we stained discs for Crb protein (Fig. 1J). Surprisingly, we do not see any significant accumulation of Crb in mutant discs compared to wild-type discs, suggesting that tumour formation can occur even when apico-basal polarity is not significantly disrupted.

A feature of neoplastic tumour suppressor genes is that clones of mutant cells surrounded by wild-type tissue do not overgrow, and in fact often proliferate less than the surrounding tissue (Agrawal et al., 1995; Brumby and Richardson, 2003; Igaki et al., 2006, 2009; Lu and Bilder, 2005; Ohsawa et al., 2011; Uhlirova et al., 2005). We generated mitotic clones of *Rbpn-5* in *Drosophila* pupal wings using the FLP/FRT system and *Ubx-FLP. Rbpn-5* mutant clones are viable and behave in a very similar way to wild-type cells, neither obviously over- nor under-proliferating. Moreover, no defects were seen in these clones compared to wild-type tissue when stained with a series of markers (Fig. 1K-L and not shown). In summary, these results suggest that *Rbpn-5* is a novel neoplastic tumour suppressor gene that displays both similarities to and differences from neoplastic tumour suppressor genes already characterised.

### *Drosophila* Rbpn-5 binds to Rab5

**Fig. 2:**
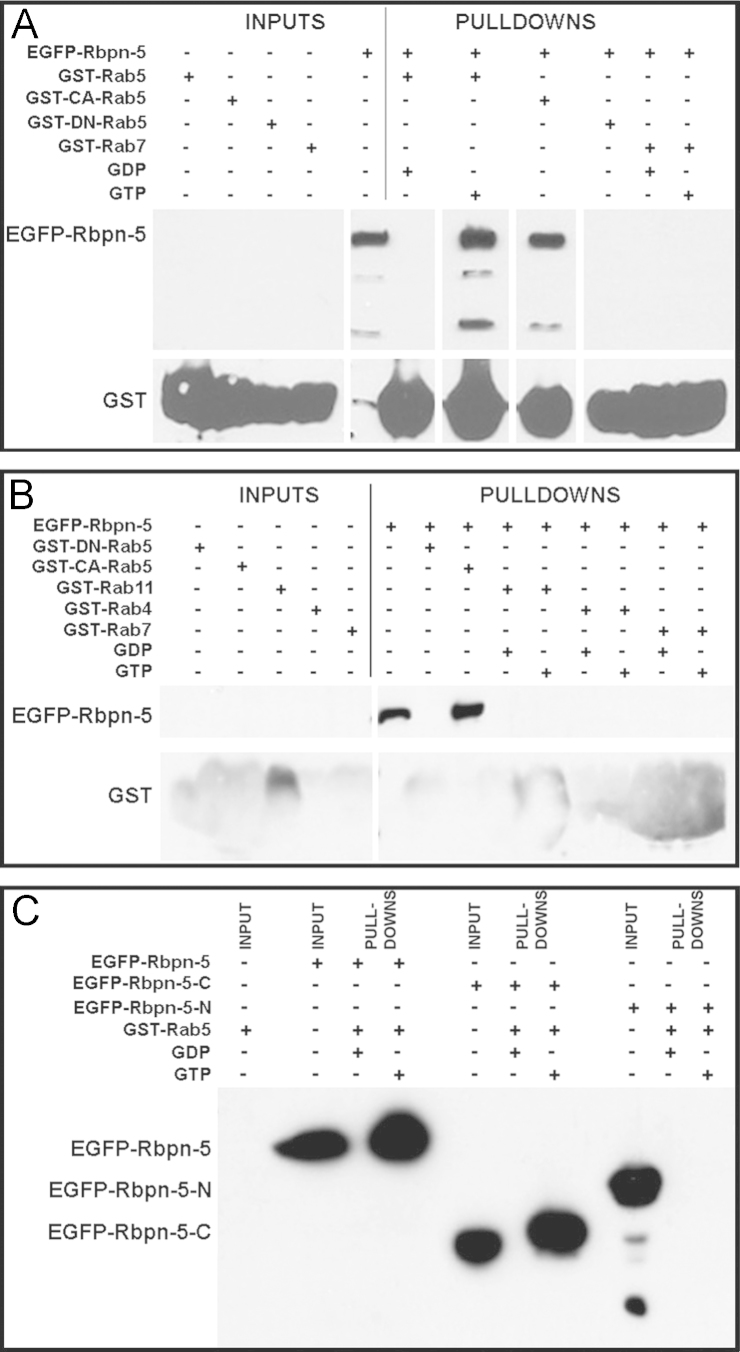
Rbpn-5 binds to Rab5 in a GTP-dependent manner. GST pulldowns showing binding of EGFP-Rbpn-5 to GST-tagged Rab proteins on a Gluthathione sepharose column (see Section Materials and Methods for details). (A,B) EGFP-Rbpn-5 binds specifically to GST-Rab5 in the presence of GTP, or to GST-*CA*-Rab5, a constitutively active form of the protein, but not to other GST-Rab proteins including Rab4, Rab7 and Rab11 or to a dominant-negative Rab5 (GST-DN-Rab5). Blots were probed with anti-Rbpn-5, which recognises both Rbpn-5 and (weakly) GST. Smaller bands in A are variably present and may represent the endogenous Rbpn-5 protein and/or degradation products that are also pulled down (Rbpn-5 is known to undergo caspase-dependent cleavage, Cosulich et al., 1997; Swanton et al., 1999). GST bands appear swollen due to massive amounts of GST-tagged protein on beads loaded directly onto the gel. The vertical white lines in A indicate empty lanes that were removed from the image for clarity. (C) Full-length Rbpn-5 (EGFP-Rbpn-5) or the C-terminal half of Rbpn-5 (EGFP-Rbpn-5-C) bind to GST-Rab5 plus GTP, but the N-terminal half of Rbpn-5 (EGFP-Rbpn-5-N) does not. The blot was probed with anti-GFP, which only recognises EGFP-tagged protein. Note there appears to be a slight shift in the size of EGFP-Rbpn-5-C when pulled down by activated Rab5. Smaller bands in the EGFP-Rbpn-5-N lane probably represent degradation products.

Mammalian Rabaptin-5 is considered to be an effector of Rab5 as it binds to it in a GTP-dependent manner in order to mediate certain functions including Rab5 recruitment and early endosome fusion (Stenmark et al., 1995). To test whether this function might be conserved in *Drosophila*, we carried out GST pulldowns to look for binding between Rbpn-5 and various *Drosophila* Rab GTPases. We found that as in vertebrates, *Drosophila* Rbpn-5 binds to Rab5 in the presence of GTP, or to an activated form of Rab5, but does not bind to Rab5 in the presence of GDP or to a dominant-negative form of Rab5 (Fig. 2A-B). To test where in the Rbpn-5 protein Rab5 might bind, we split Rbpn-5 into N- and C-terminal fragments (see Materials and Methods) and found as expected that Rab5 binds to the C-terminal part of Rbpn-5 (containing the predicted Rab5-binding domain) and not to the N-terminal fragment (Fig. 2C). We did not observe binding of Rbpn-5 to any other Rab protein tested including Rab4, Rab7, Rab11, Rab8 or Rab23 (Fig. 2B and data not shown). The absence of binding to Rab4 is surprising, since mammalian Rabaptin-5 does bind to Rab4 in a GTP-dependent manner to provide a link between endocytosis and the fast recycling pathway (Daro et al., 1996; Deneka et al., 2003; Pagano et al., 2004; van der Sluijs et al., 1992; Vitale et al., 1998). The putative Rab4 binding domain is in the N-terminal half of Rabaptin-5 (Vitale et al., 1998), which appears to be less highly conserved in invertebrates. This result mirrors that seen with Rbsn-5, another Rab5 effector whose mammalian homologue binds to Rab4 in vertebrate cells, but whose *Drosophila* homologue does not (de Renzis et al., 2002; Morrison et al., 2008; Nielsen et al., 2000), suggesting that the fast recycling pathway may not be regulated in the same manner in *Drosophila* as it is in mammalian cells.

### *Drosophila* Rbpn-5 modulates early endosome dynamics *in vivo*

**Fig. 3:**
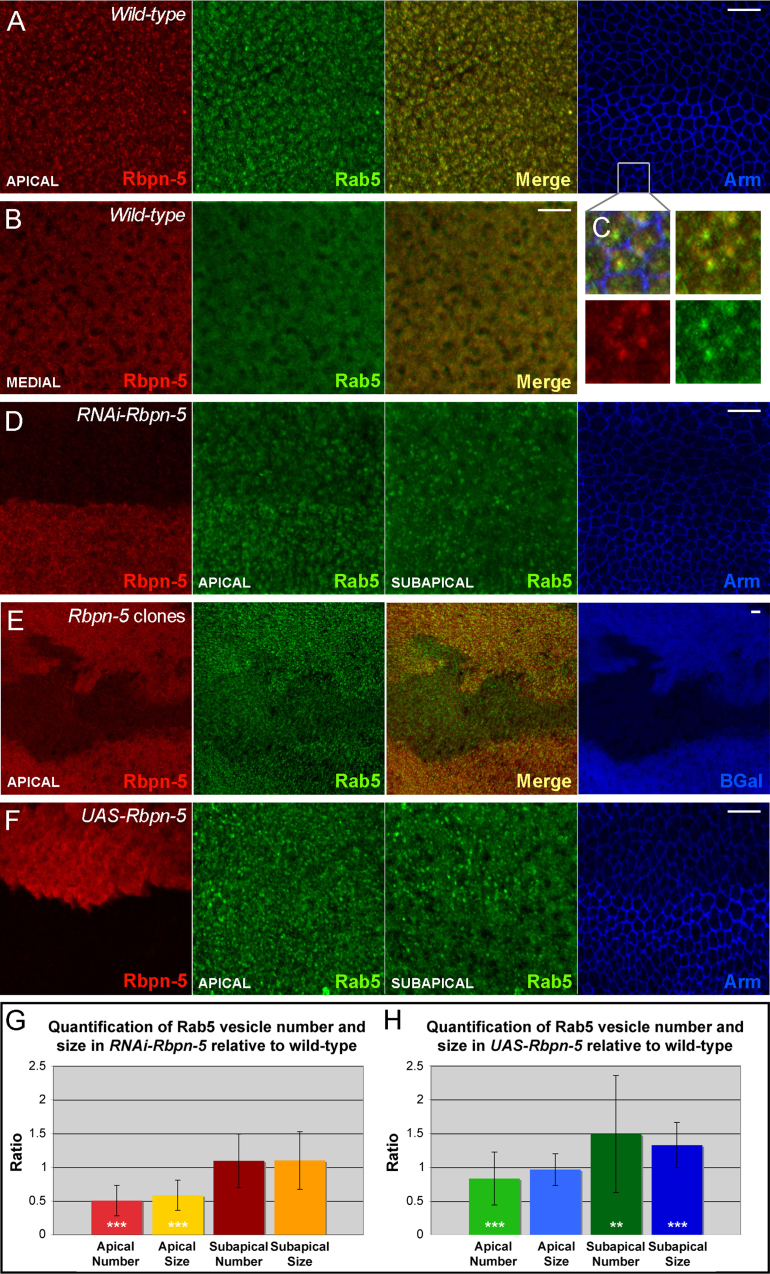
Rbpn-5 modulates early endosome dynamics in the *Drosophila* wing. (A–C) Rbpn-5 co-localises with Rab5 in *Drosophila* early pupal wings. Apically, Rbpn-5 colocalises with Rab5 in puncta, suggesting that Rbpn-5 localises to early endosomes (A, zoom in C). Medially (B), Rbpn-5 and Rab5 are predominantly cytoplasmic. Rab5 levels are reduced with little punctate staining and little co-localisation is seen. (D) Expression of *RNAi-Rbpn-5* almost eliminates Rbpn-5 staining and strongly reduces apical Rab5 puncta (top half of each image). Subapical Rab5 puncta are not strongly affected. (E) *Rbpn-5* mutant *Ubx-FLP* generated clones (marked by an absence of β-Gal in blue) show a similar reduction in Rbpn-5 staining and loss of apical Rab5 puncta. (F) Over-expressing Rbpn-5 results in a slight increase in number and size of subapical Rab5 puncta (top half of each image). Apical Rab5 puncta are slightly reduced. In all images (A–F) distal is to the right and anterior is upwards. Rbpn-5 staining is marked in red and Rab5 staining in green. Apart from E, images show an area distal to the posterior crossvein, with the L3 longitudinal wing vein extending horizontally along the centre of the image. UAS and RNAi constructs are expressed with the *ptc-Gal4* driver above L3 (top half of the image), whilst tissue below L3 is wild-type (lower half of the image). *UAS-Dicer2* was co-expressed with RNAi constructs to enhance efficacy. Wings were dissected at 4.5 h APF at 29 °C. Scale bars are 5 μm long. (G-H) Quantification of Rab5 vesicle number and size. Quantification and statistical analysis was carried out as described in the Materials and Methods. Mean apical and subapical values for vesicle number and size were normalised to wild-type before plotting on a graph. Stars indicate statistical difference between wild-type and mutants calculated from the raw data using paired t-tests. Three stars=*p*<0.001 and two stars=*p*<0.01. (H) Quantification of *RNAi-Rbpn-5* expressing tissue relative to wild-type shows that apical puncta are significantly reduced in both number and size. (I) Quantification of *UAS-Rbpn-5* expressing tissue relative to wild-type shows that subapical vesicles are significantly increased in both number and size, but that slightly fewer apical vesicles are also present.

We raised an antibody against *Drosophila* Rbpn-5 (see Materials and Methods) and observed its localisation in pupal wings. Rbpn-5 is uniformly expressed and localises to punctate apical structures together with some cytoplasmic staining deeper within the cell (Fig. 3A--C). The punctate staining co-localises well with an antibody against Rab5, indicating that these vesicles are probably early endosomes. As previously found (Mottola et al., 2010), Rab5 puncta appear to be enriched apically: this is most striking in early pupal wings (4.5 h after puparium formation at 29 °C), but can also be observed in third instar larval wing discs and later stage pupal wings (26 h APF at 29 °C, Fig. 3A-B and data not shown).

Use of the *patched-GAL4* (*ptc-GAL4*) driver to express *RNAi-Rbpn-5* in a stripe anterior to the L4 wing vein, indicated that RNAi using either dsRNA construct is extremely effective in reducing the levels of Rbpn-5 protein (Fig. 3D and data not shown). Interestingly, RNAi expression significantly reduces the number and size of apical Rab5 puncta, but has no effect on puncta observed just subapical to the surface (~0.5 μm below, Fig. 3D,G). A similar effect is seen in *Rbpn-5* loss-of-function clones (Fig. 3E). This suggests that Rbpn-5 is involved in promoting apical localisation of Rab5 and hence is probably involved in regulating Rab5-mediated endocytosis from the apical surface.

We also made constructs to over-express Rbpn-5 under control of the UAS promoter. In mammalian cells, it has been shown that transfection of Rabaptin-5 results in excessive homotypic early endosome fusion, resulting in a characteristic enlarged early endosome phenotype (Stenmark et al., 1995). We observed a much more subtle phenotype in the *Drosophila* wing, whereby the number and size of subapical early endosomes is slightly, but significantly increased (Fig. 3F,H). However, we did also observe a slight reduction in the numbers of apical early endosomes. These results taken together suggest that in *Drosophila*, Rbpn-5 is necessary to promote normal levels of early endosome fusion, but is not sufficient to enhance the process by more than a very small amount, possibly due to limiting quantities of other proteins involved in early endosome fusion.

**Fig. S3:**
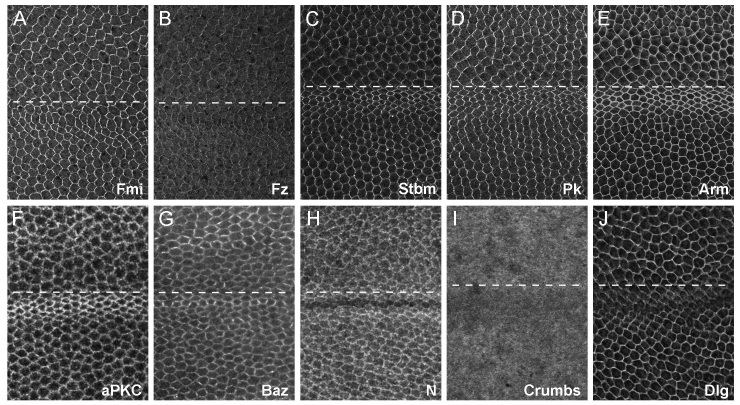
Localisation of markers in *RNAi-Rbpn-5* expressing pupal wings. All markers analysed show a wild-type distribution when *RNAi-Rbpn-5* is expressed using the *ptc-Gal4* driver. Images show the L4 vein in the centre (just below the dotted line), with the *ptc-Gal4*-expressing domain above the dotted line and wild-type tissue below the dotted line. (A–D) Core planar polarity proteins Fmi (A), Fz (B), Stbm (C) and Prickle (Pk, D) localise to junctions at wild-type levels. Asymmetry is slightly disrupted in RNAi-expressing tissue and cell packing appears slightly abnormal. However, similar defects were seen with control RNAi expression. No alteration in other markers is seen; (E) Arm, (F) aPKC, (G) Baz, (H) N, (I) Crb and (J) Dlg. All images show *GD26367 RNAi-Rbpn-5* crossed to *ptc-Gal4* with *UAS-Dcr2*, but similar results were obtained with *pWIZ RNAi-Rbpn-5* and without *UAS-Dcr2*. Pupae were aged at 29 °C for 26 h before dissection. In all wings anterior is up and distal is to the right.

If a lack of Rbpn-5 significantly influences early endosome dynamics, then we would expect to see defects in the localisation of proteins that are normally endocytosed through this pathway. To this end, we examined a large number of markers in pupal wings expressing *RNAi-Rbpn-5* under the *ptc-Gal4* driver and compared them to adjacent wild-type cells. However, we were unable to find significant differences in localisation of any protein examined including those involved in apico-basal polarity or planar polarity, either in early or late pupal wings (Fig. S3 and data not shown). This suggests that despite the reduction in apical Rab5-positive vesicles, a sufficient level of endocytosis for normal function is still present in *RNAi-Rbpn-5* expressing tissue.

### *Drosophila* Rbpn-5 and Rabex-5 interact to modulate early endosome dynamics

**Fig. 4:**
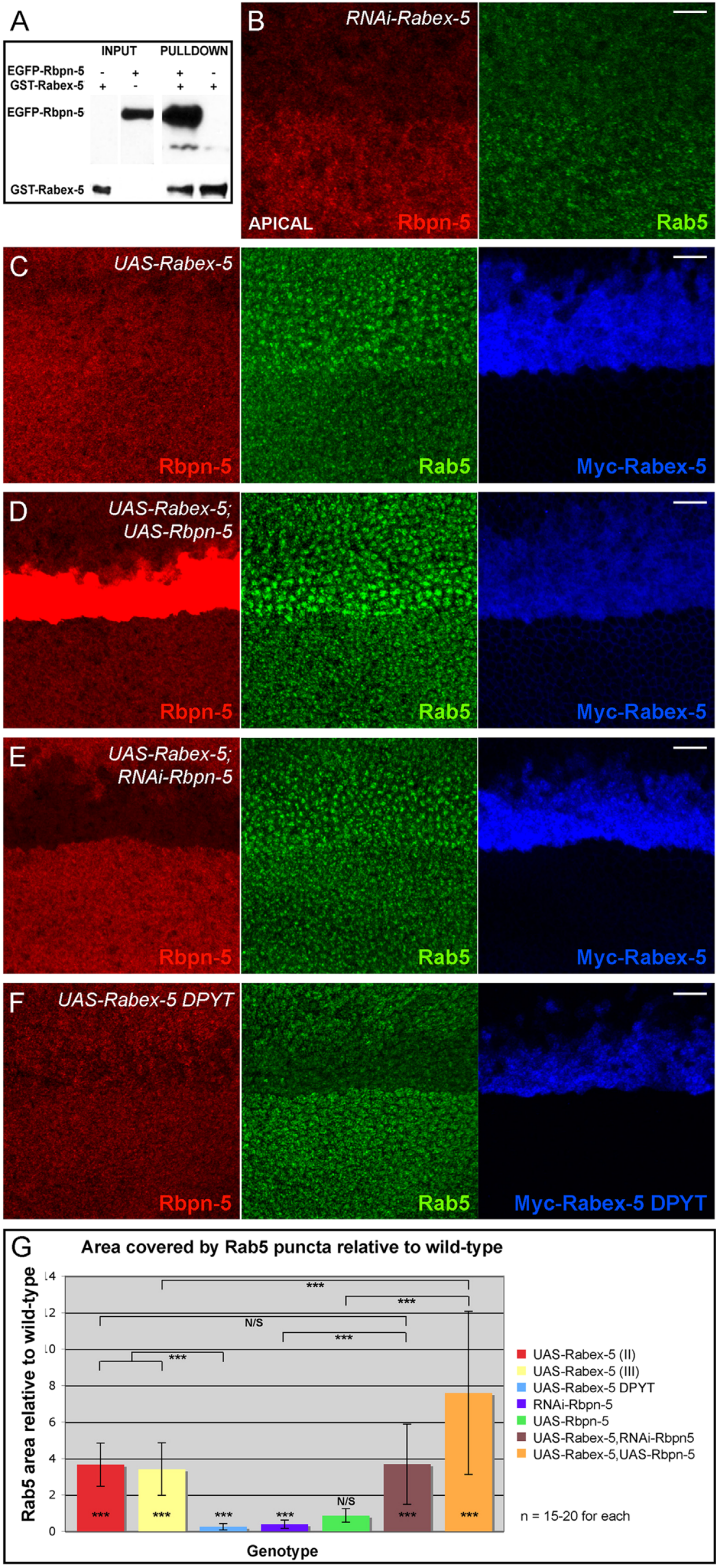
Rbpn-5 binds to Rabex-5 and together they modulate early endosome dynamics *in vivo*. (A) GST pulldown showing binding of EGFP-Rbpn-5 to GST-Rabex-5 on a Gluthathione agarose column (see Section Materials and Methods for details). The blot was probed with anti-Rbpn5, which recognises EGFP-Rbpn-5 and (weakly) GST. (B) Expression of *RNAi-Rabex-5* strongly reduces Rbpn-5 staining and apical Rab5 puncta (top half of each image). (C) Over-expressing Rabex-5 results in a strong increase in Rab5 positive vesicles, suggesting that Rabex-5 promotes early endosome fusion. (D) Co-expression of Rabex-5 and Rbpn-5 causes a synergistic increase in Rab5 recruitment to puncta. (E) Depleting Rbpn-5 activity using RNAi does not reduce the ability of excess Rabex-5 to recruit Rab5 to puncta. (F) Expression of a mutated Rabex-5 that lacks GEF activity causes a strong reduction in Rab5 puncta, indicating that Rab5 recruitment is dependent upon the GEF activity of Rabex-5. Images B–F are presented as described for Fig. 3 and scale bars are 10 μm long in all. The over-expression of Myc-tagged Rabex-5 constructs with *ptc-Gal4* was verified by anti-Myc staining in blue. (G) Quantification of Rab5 puncta in the above genotypes. Quantification and statistical analysis was carried out as described in Section Materials and Methods. The value of puncta area is a factor of both puncta number and size. Mean values for puncta area were normalised to wild-type before plotting on a graph. Stars on bars indicate statistical difference between wild-type and mutants calculated from the raw data using paired t-tests, whereas stars above two bars represent statistical difference between two mutants, calculated from normalised data using an unpaired t-test. Three stars=*p*<0.001.

In vertebrates, Rabaptin-5 acts to recruit activated Rab5 to endocytic sites through binding to and enhancing the activity of Rabex-5, a GTP exchange factor (GEF) that converts inactive GDP bound Rab5 to the active GTP bound form (Esters et al., 2001; Horiuchi et al., 1997). To test whether this interaction might be conserved in *Drosophila*, we carried out GST pulldowns between Rbpn-5 and the *Drosophila* homologue of Rabex-5, and found that the two proteins interact physically (Fig. 4A). Mammalian Rabex-5 binds to a coiled-coil region in the third quarter of Rabaptin-5 (Mattera et al., 2006), which has homology to the central region of Rbpn-5 (Fig. 1A). Since this domain is split between the N- and C-terminal halves of Rbpn-5 that we previously generated, we did not anticipate that Rabex-5 would be able to pull down either half individually, and indeed we could not demonstrate binding to either fragment (data not shown).

*In vitro* assays have shown that Rabaptin-5 requires Rabex-5 both for its recruitment to early endosomes and its ability to promote early endosome fusion, whereas Rabex-5 alone can stimulate excessive early endosome fusion if expressed at high enough levels (Horiuchi et al., 1997; Lippe et al., 2001). We obtained *RNAi* and *UAS* lines to examine the effect of Rabex-5 on early endosome dynamics in the *Drosophila* wing (VDRC and Yan et al., 2010). As with *RNAi-Rbpn-5*, apical Rab5 puncta are significantly reduced in *RNAi-Rabex-5* expressing tissue (Fig. 4B). We also observed reduced levels of Rbpn-5 (Fig. 4B), suggesting that as *in vitro*, Rabex-5 is required to recruit Rbpn-5 to apical endosomes in *Drosophila*. Conversely, over-expression of *Rabex-5* causes a large increase in both size and number of apical and sub-apical early endosomes (Fig. 4C,G). Furthermore, co-expression of *UAS-Rbpn-5* enhances this phenotype (Fig. 4D,G), indicating that the two proteins can act together synergistically to promote early endosome fusion, and suggesting that the inability of *UAS-Rbpn-5* to have much effect alone may be due to limiting levels of Rabex-5 normally present in the *Drosophila* wing. To test the requirement for Rbpn-5 in Rabex-5-mediated early endosome fusion, we removed Rbpn-5 function using RNAi, whilst also expressing ectopic Rabex-5. Enlargement of early endosomes is still seen to a similar level as with *UAS-Rabex-5* expression alone (Fig. 4E,G), indicating that excess Rabex-5 has the ability to promote early endosome fusion even when Rbpn-5 is depleted. We also expressed a mutant version of Rabex-5 that lacks GEF activity, *UAS-Rabex-5*^*DPYT*^ (Yan et al., 2010). As expected, no increase in early endosome size or number is observed and in fact levels of apical Rab5 puncta are severely reduced (Fig. 4F), indicating that Rab5 recruitment and early endosome fusion are dependent upon the GEF activity of Rabex-5. These results are in agreement with previous work from tissue culture (Horiuchi et al., 1997; Lippe et al., 2001), and suggest that the Rbpn-5/Rabex-5 complex is likely to be physically and functionally conserved in *Drosophila*.

### *Rabex-5* is a neoplastic tumour suppressor gene

A *Rabex-5* hypomorphic mutant has previously been characterised and shown to form giant larvae or pupae, which often contain melanotic tumours (Yan et al., 2010). Through several pieces of evidence, these phenotypes have been attributed to misregulation of Ras signalling, which is known to promote growth and cause melanotic tumours (Yan et al., 2010; Zettervall et al., 2004). Rabex-5 regulates Ras signalling not through its GEF activity, but through a separate ubiquitin ligase domain (Mattera et al., 2006; Xu et al., 2010; Yan et al., 2010), although since ubiquitinated cargo including Ras are then targeted to early endosomes, in practice the two functions of the protein are likely to be highly interdependent (Aikawa, 2012; Aikawa et al., 2012; Mattera and Bonifacino, 2008).

**Fig. S4:**
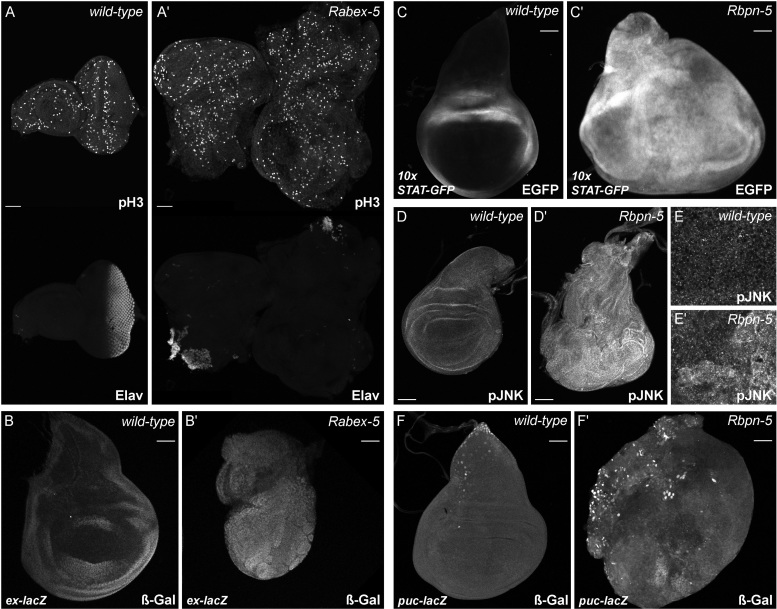
Expression of markers in Rabex-5 and Rbpn-5 neoplastic discs. Expression of various markers in wild-type (A–F) and *Rabex-5* (A′–B′) or *Rbpn-5* (C′–F′) mutant discs. In all discs (where possible to tell), anterior is to the left and dorsal is upwards. Scale bars are 50 μm long. (A) anti-histone H3 (phospho S10) is a mitosis marker. Wild-type eye discs (A) show a down-regulation of cell division within the morphogenetic furrow, a row of mitotic cells just posterior to the furrow and a strong reduction of cell division in differentiated ommatidia as shown by Elav staining. Cell division is not down-regulated in *Rabex-5* mutant eye discs and this correlates with strongly impaired differentiation, excessive overgrowth, and delayed pupation. (B) *ex-lacZ* is a read-out for the Hpo/Wts pathway activity (Boedigheimer and Laughon, 1993). Both wild-type (B) and *Rabex-5* mutant (B′) wing discs show a broad distribution of *ex-lacZ* staining at similar levels. The extent of the expression domain in wild-type discs and the lack of clear disc domains in non-differentiating mutant discs makes it difficult to ascertain whether *ex-lacZ* is increased or remains the same in the *Rabex-5* mutant. (C) *10xSTAT-GFP* is a read-out for JAK/STAT pathway activity (Bach et al., 2007). In wild-type wing discs, a ring of 10xSTAT-GFP is seen around the wing pouch, in the region corresponding to the wing hinge (C). Massive levels of *10xSTAT-GFP* are observed throughout *Rbpn-5* mutant wing discs (C′). (D–E) In wild-type wing discs, activated JNK (pJNK) is present at fairly low levels throughout (D–E), whereas in *Rbpn-5* mutant wing discs, pJNK levels are increased (D′–E′). E is a magnification of part of the wing pouch area, showing pJNK accumulating in puncta. (F) *puc*^*E69*^ (*puc-lacZ*) is a read-out for the JNK pathway activity (Martín-Blanco et al., 1998). In wild-type wing discs (F) *puc-lacZ* is activated strongly only at the distal tip and in a few cells of the presumptive notum, whereas in *Rbpn-5* mutant discs (F′), patches of ectopic high level *puc-lacZ* expression are seen, together with a general level of upregulation throughout the disc. Mutant discs are highly variable in their level of *puc-lacZ* expression, with some showing extremely high expression throughout the disc and others showing little more than wild-type.

**Fig. 5:**
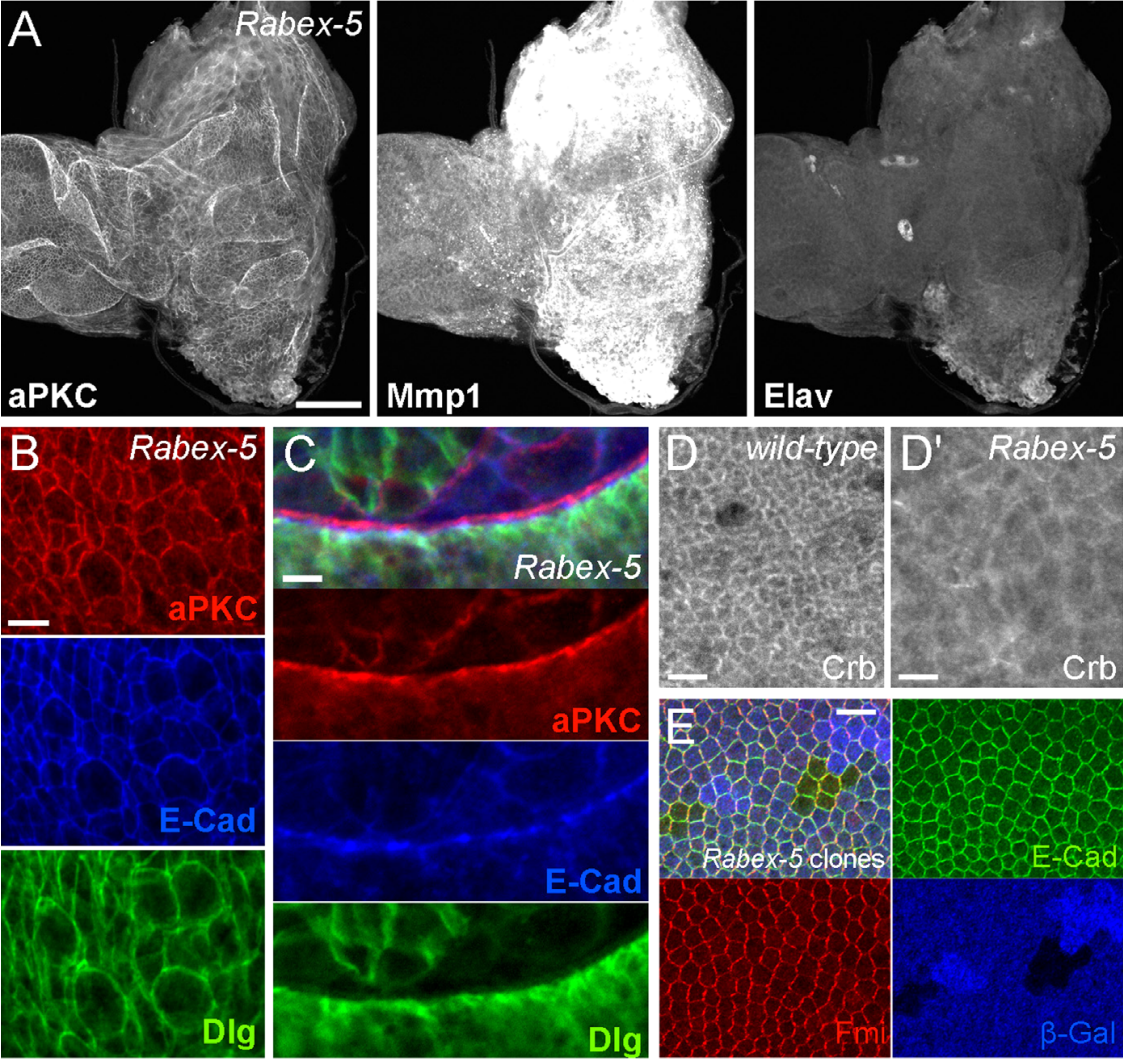
*Rabex-5* is a neoplastic tumour suppressor gene. (A) *Rabex-5*/*Df(3L)BSC250* eye discs stained for aPKC, Mmp1 and Elav. Differentiation is absent and Mmp1 levels are highly upregulated. Anterior is to the left. Scale bar is 50 μm long. (B-C) Wing discs stained with aPKC in red (apical), E-Cad in blue (junctional) and Dlg in green (baso-lateral) in *Rabex-5* mutants. As seen in *Rbpn-5*, cells have a larger apical surface (B) but apico-basal polarity markers localise correctly at cell edges and show clear separation in a lateral view (C). Scale bars are 5 μm long. (D) Wild-type (D) and *Rabex-5* mutant (D′) wing discs stained for Crb. Laser power and gain was kept at the same level for both images. Scale bars are 5 μm long. (E) Localisation of markers is normal in *Rabex-5* mutant clones visualised in wings at 28 h APF. E-Cad is green and Fmi is red. Clones are marked by absence of β-Gal staining (blue). Scale bar is 10 μm long.

Although Ras signalling promotes hyperplastic growth rather than neoplastic growth, the similarity of the described *Rabex-5* mutant phenotype to that of other endocytic tumour suppressor genes led us to investigate the mutant animals more closely. As previously described (Yan et al., 2010), we observed that *Rabex-5* homozygous or *Rabex-5*/*Df(3L)BSC250* transheterozygous mutant larvae grow excessively due to delayed pupation, and contain occasional melanotic tumours (data not shown). We examined imaginal discs and found that as for *Rbpn-5*, the disc epithelium of *Rabex-5* mutants is over-grown and folded, cell size is increased and tissue labels strongly for Mmp1 expression (Fig. 5A-B). Eye-antennal discs are often fused with the brain lobes, Elav staining is variable and sometimes completely absent (*e.g.* Fig. 5A), and cell division is no longer clearly regulated (Fig. S4A), indicating that differentiation is compromised. However, again apico-basal polarity appears to be maintained in *Rabex-5* mutants, since clear separation of apical, junctional and baso-lateral markers is seen and Crb levels are not increased compared with wild-type (Fig. 5B–D). *Ubx-FLP*-induced clones lacking *Rabex-5* function were also generated. Cells within clones do not over-proliferate compared to twin-spots, and tissue appears wild-type with respect to a range of markers analysed (Fig. 5E and not shown). Together, these results indicate that *Rabex-5* is a neoplastic tumour suppressor gene.

### JNK and JAK/STAT pathways are upregulated in *Rabex-5* neoplastic tumours

*Drosophila* neoplastic tumour phenotypes have been attributed to several mutually non-exclusive mechanisms. Mis-regulation of apico-basal polarity due to apical accumulation of Crb protein has been proposed as key step in tumourigenesis in early endocytic mutants (Lu and Bilder, 2005; Moberg et al., 2005; Vaccari and Bilder, 2009). Excess Crb is able to activate the Hippo (Hpo)/Warts (Wts) signalling pathway, thus promoting growth (Chen et al., 2010; Grzeschik et al., 2010; Ling et al., 2010; Robinson et al., 2010; Robinson and Moberg, 2011). However, neither *Rbpn-5* nor *Rabex-5* mutant discs exhibit apico-basal polarity defects, and Crb does not significantly accumulate (Figs. 1F–J, 5B–D), indicating that this mechanism is not obligatory for neoplastic tumour formation. Hpo/Wts signalling is active throughout mutant discs as shown by an *expanded-lacZ* (*ex-lacZ*) reporter (Fig. S4B, Boedigheimer and Laughon, 1993), suggesting that this pathway might be contributing to disc growth. However, the effects are relatively mild and it is difficult to ascertain whether the broader expression domain compared to wild-type discs is significant or simply a side-affect of impaired differentiation.

**Fig. 6:**
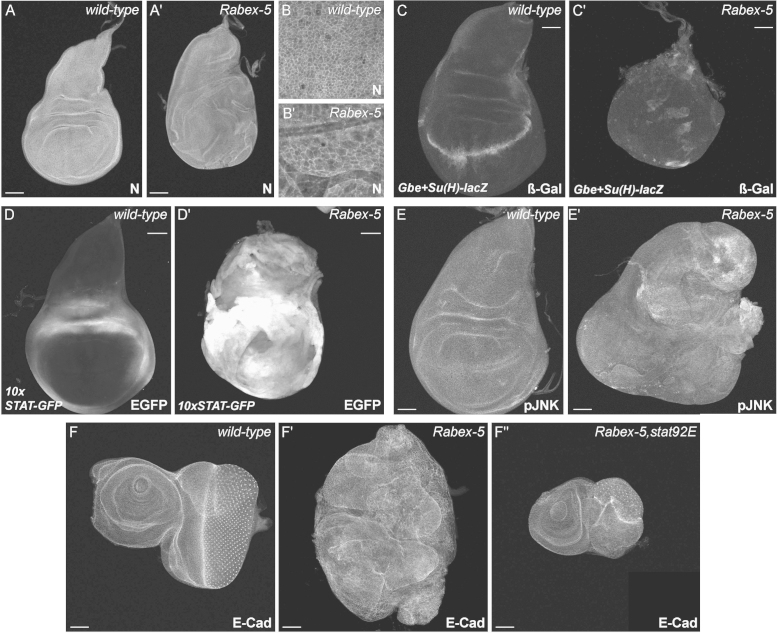
Rabex-5 neoplastic tumours show increased JNK and JAK/STAT pathway activity. Expression of various signalling pathway reporter genes and components in wild-type (A–E) and *Rabex-5* mutant (A′–E′) wing discs. In all discs (where possible to tell), anterior is to the left and dorsal is upwards. Scale bars are 50 μm long. (A-B) N protein localises at a similar intensity to apical junctions and intracellular puncta in both wild-type and *Rabex-5* mutant wing discs. (B) shows magnification of wing pouch area. (C) *Gbe+Su(H)-lacZ* expression is a read-out for N pathway activity (Furriols and Bray, 2001). A stripe of N activation is seen at the dorso-ventral boundary of the wild-type wing pouch (C), but no increase in N activity is observable in the *Rabex-5* mutant wing disc (C′). (D) *10xSTAT-GFP* is a read-out for the JAK/STAT pathway activity (Bach et al., 2007). In wild-type wing discs, a ring of 10xSTAT-GFP is seen around the wing pouch, in the region corresponding to the wing hinge (D). Massive levels of *10xSTAT-GFP* are observed in *Rabex-5* mutant wing discs (D′). (E) In wild-type wing discs, activated JNK (pJNK) is present at fairly low levels throughout (E), whereas in *Rabex-5* mutant wing discs, pJNK levels are increased (E′). (F) A hypomorphic mutation in *stat92E* rescues various aspects of the neoplastic tumour phenotype as demonstrated by E-Cad staining. Unlike *Rabex-5* single mutant eye discs (F′), *Rabex-5*, *stat92E* double mutant eye discs do not invade the brain, are not overgrown or misshapen and ommatidial differentiation occurs in a fairly wild-type fashion, but tissue is still abnormally folded (F″).

In addition to Crb, other molecules have been found to accumulate apically in endocytic mutants, and ectopic activity of many signalling pathways has been documented in neoplastic tumours, including N, JNK and JAK/STAT. In order to try to investigate the mechanism of the neoplastic tumour phenotypes in more detail, we decided to look at the expression of a range of markers for activation of different signalling pathways. N has been shown to accumulate apically in early endocytic mutants and in subapical vesicles in ESCRT mutants, with only the latter resulting in N pathway activation (Herz et al., 2006,2009; Lu and Bilder, 2005; Moberg et al., 2005; Morrison et al., 2008; Thompson et al., 2005; Vaccari and Bilder, 2005; Vaccari et al., 2008). ESCRT mutant clones display a non-autonomous phenotype whereby ectopic N signalling promotes expression of the JAK/STAT ligand Upd, which then promotes growth in adjacent wild-type cells (Herz et al., 2006; Moberg et al., 2005; Vaccari and Bilder, 2005). We do not observe N accumulation in either *Rbpn-5* or *Rabex-5* mutant discs (Figs. 6A-B, S2C), and unsurprisingly, no increase is seen in expression of the N reporter *Gbe-Su(H)-lacZ* (Furriols and Bray, 2001, Fig. 6C), indicating that the N pathway is not ectopically activated. However, we do observe massive upregulation of JAK/STAT pathway activity in both mutants as assayed using a *10xSTAT-GFP* reporter line (Bach et al., 2007, Figs. 6D, S4C).

In order to test whether JAK/STAT signalling plays an instructive role in tumourigenesis, we used a hypomorphic *stat92E* mutation (*Frankenstein*—*stat92E*^*F*^) to try to rescue the tumour phenotype of *Rabex-5* mutant discs. *stat92E*^*F*^ homozygotes are viable as adults, but produce knobbly outgrowths on the dorsal prothorax (Baksa et al., 2002 and data not shown). We found that like *Rabex-5* single mutants, *Rabex-5*, *stat92E*^*F*^ homozygotes die during pupation, but there is no over-growth of either larvae or imaginal discs (Fig. 6F and data not shown). The tumourous phenotype of double mutant discs is significantly milder than that of single mutants, as eye-antennal discs are no longer fused to the brain, but show a relatively wild-type shape and structure, together with clear signs of differentation (Fig. 6F). However, abnormal folds are still observed suggesting that not all aspects of tumourigenesis are downstream consequences of ectopic JAK/STAT pathway activity.

As *Rabex-5* and *Rbpn-5* mutants both show upregulation of the JNK target gene Mmp1 (Figs. 1E, 5A), it appeared likely that ectopic activation of the JNK pathway could also be contributing to tumour formation. We tested this using an antibody against activated (phosphorylated) JNK (pJNK) and indeed found a patchy accumulation of protein in *Rabex-5* and *Rbpn-5* mutant discs (Figs. 6E, S4D-E). In addition, we observed a variable increase in expression of the JNK *lac-Z* reporter *puc*^*E69*^ (Martín-Blanco et al., 1998, Fig. S4F), suggesting that there is a significant upregulation of the JNK pathway in mutant discs, although it does not appear to be as dramatic as ectopic activation of the JAK/STAT pathway.

In summary, these results suggest that the neoplastic tumour suppressor phenotypes are linked to activation of JNK and JAK/STAT pathways, but are not caused by defective N signalling, or disrupted apico-basal polarity.

## Discussion

### *Rbpn-5* and *Rabex-5* are novel tumour suppressor genes

Endocytosis is increasingly recognised as an important mechanism for the growth and metastasis of tumours, both in the clinical setting and in cancer models. The categorisation of endocytic neoplastic tumour suppressor genes in *Drosophila* began less than ten years ago with the identification of *Rab5*, *avl*, *Vps25* and *Tsg101* (Lu and Bilder, 2005; Moberg et al., 2005; Thompson et al., 2005; Vaccari and Bilder, 2005) and has since expanded with the discovery of at least 14 others, in particular multiple components of the ESCRT complex (Herz et al., 2009; Menut et al., 2007; Morrison et al., 2008; Rodahl et al., 2009; Vaccari et al., 2009). Most mutations so far identified are zygotic lethal long before the third instar larval stage and so have been discovered through screens in which homozygous mutant clones were generated that spanned entire epithelial compartments in otherwise heterozygous animals. However, not all chromosome arms have been screened and screens have not been saturating, hence it is likely that many others remain to be found.

In this study we identify two novel endocytic neoplastic tumour suppressor genes. *Rbpn-5* has not been previously characterised in *Drosophila*, and our work is the first to show that not only does it act as a neoplastic tumour suppressor gene in *Drosophila*, but that its endocytic functions, initially investigated in mammalian cell culture and *in vitro* (Stenmark et al., 1995), are conserved in a multi-cellular organism during development. Unlike many of the *Drosophila* endocytic neoplastic tumour suppressor genes so far identified, *Rbpn-5* has a human homologue that has been directly implicated in tumour growth and metastasis. Mammalian Rabaptin-5 controls integrin recycling during migration of invasive tumour cells, has been identified in patients suffering from myelomonocytic leukaemia, is involved in preventing hypoxia in primary kidney and breast tumours and has been shown to interact physically with the Tuberous sclerosis protein Tuberin (Christoforides et al., 2012; Magnusson et al., 2001; Wang et al., 2009; Xiao et al., 1997). Although not all of these functions may be conserved in *Drosophila*, our work provides a basis for further investigating the mechanisms of Rbpn-5 dependent tumourigenesis in a whole animal system.

*Rabex-5* has been previously identified as a tumour suppressor gene in *Drosophila* (Yan et al., 2010), but its neoplastic characteristics were not described. Importantly, both *Rbpn-5* and *Rabex-5* mutants are homozygous viable until late larval stages, and thus may provide a more facile model than other early endocytic tumour suppressor genes for uncovering the mechanistic basis of neoplasia.

We had initially set out to identify new regulators of planar polarity through an RNAi screen. As core planar polarity proteins are known to undergo internalisation and recycling (Mottola et al., 2010; Shimada et al., 2006; Strutt and Strutt, 2008; Strutt et al., 2011), and Rbpn-5 and Rabex-5 are general endocytic regulators that are functional in epithelial tissues at the time of planar polarisation, it is highly likely that they are playing a role in trafficking of core proteins. Indeed, another Rab5 effector, Rbsn-5, which is also a neoplastic tumour suppressor protein, regulates Fmi localisation (Morrison et al., 2008; Mottola et al., 2010). Unlike Rbsn-5, cell-autonomous depletion of Rbpn-5 or Rabex-5 does not significantly alter planar polarity protein levels or localisation (Figs. 1L, 5E, S3 and data not shown), suggesting that some level of endocytosis can occur in the absence of these proteins. One possibility is that Rbpn-5 and Rabex-5 proteins might be highly stable compared with Rbsn-5. Evidence in support of this comes from zygotic mutants, which survive to early pupal stages for *Rbpn-5* and *Rabex-5* (Fig. 1B, Yan et al., 2010), presumably due to perdurance of maternal protein, but die before the second larval instar in the *Rbsn-5* background (Morrison et al., 2008). If this is the case, residual protein activity in clones or RNAi-treated tissue may allow a low level of general endocytosis to occur, which would be sufficient to correctly localise core planar polarity proteins. Another, non-mutually exclusive hypothesis is that a level of endocytosis can occur in the absence of either Rabex-5 or Rbpn-5. There are three other Rab5 GEF homologues in *Drosophila* (Smythe, unpublished data), and these may be able to step in if Rabex-5 is absent.

Whatever the reason may be, the lack of defects seen in clones or RNAi, combined with the severe pleiotropic effects on epithelial architecture observed in zygotic mutants (Figs. 1C,E, 5A, and S2), means that analysis of a specific role in planar polarity is extremely complex and we decided to focus our attentions on their tumour suppressor roles.

### The Rab5 effector function of Rabaptin-5 is conserved in *drosophila*

In mammalian cells, it has been shown that Rabaptin-5 is an effector of Rab5 that promotes early endosome fusion through its interaction with Rabex-5 (Horiuchi et al., 1997; Lippe et al., 2001; McBride et al., 1999; Stenmark et al., 1995; Vitale et al., 1998). We provide here the first characterisation of a Rabaptin-5 homologue in a multicellular organism, and show that many of its functions are conserved. Rab5 effectors are defined by several criteria including GTP-dependent binding to Rab5 and the ability to modulate a Rab5-dependent process in response to Rab5 activity. We show here that *Drosophila* Rbpn-5 fulfils the criteria of a Rab5 effector protein in the same way as its mammalian counterpart. Firstly we have demonstrated that Rbpn-5 physically interacts specifically with GTP-bound Rab5, probably through its predicted C-terminal Rab5 binding domain. Secondly, that Rbpn-5 and Rab5 colocalise in *Drosophila* pupal wings. Thirdly, when we deplete Rbpn-5 using RNAi or in mutant clones, we see a strong reduction in apical Rab5-positive vesicles indicating that Rbpn-5 is required for Rab5 recruitment. Lastly, if we over-express Rbpn-5 we observe subtle alterations in early endosome structure, which are indicative of a role for Rbpn-5 in promoting early endosome fusion.

Although the Rab5 effector function of Rabaptin-5 is conserved in *Drosophila*, its Rab4 effector function may not be. Mammalian Rabaptin-5 has been shown to bind Rab4 through a separate N-terminal domain, and the divalent Rab5-Rab4 binding ability allows Rbpn-5 to act as a bridge between the endocytic and fast recycling pathways (Daro et al., 1996; van der Sluijs et al., 1992; Vitale et al., 1998). We were not able to recapitulate Rab4 binding, and indeed the N-terminal Rab4 binding site is not highly conserved in *Drosophila* Rbpn-5. A similar result was found for the *Drosophila* homologue of another Rab5-Rab4 divalent effector, Rabenosyn-5 (Morrison et al., 2008), suggesting that the mechanism of transfer of cargo from the endocytic to recycling routes might not be conserved across phyla.

### The interaction between Rabaptin-5 and Rabex-5 is conserved in *drosophila*

A large number of proteins have been shown to bind Rabaptin-5, but one of the best characterised is Rabex-5. The endogenous GEF activity of Rabex-5 is fairly low, and unless large quantities are overexpressed, the formation of a Rabaptin-5/Rabex5 complex is necessary to promote nucleotide exchange on Rab5 (Esters et al., 2001; Horiuchi et al., 1997; Lippe et al., 2001; Zhu et al., 2007). The effect of this is to enhance Rab5 activity, thus facilitating its recruitment of factors involved in tethering and fusion of early endocytic membranes.

Here we provide evidence that the interaction between Rabex-5 and Rbpn-5, and their ability to alter early endosome dynamics is conserved in *Drosophila*. As with their mammalian counterparts, the two proteins bind *in vitro*: we suspect that Rabex-5 binds to a central domain in Rbpn-5, as this is homologous to the Rabex-5 binding site in mammalian Rabaptin-5 (Mattera et al., 2006), and neither N- nor C-termini of Rbpn-5 can bind alone. As in mammalian cells (Horiuchi et al., 1997; Zhu et al., 2007), over-expression of Rabex-5 in *Drosophila* wings is sufficient to promote early endosome fusion even if Rbpn-5 levels are depleted by RNAi. However, co-expression of Rbpn-5 significantly enhances this, demonstrating that the complex acts synergistically. The dependency on Rabex-5 for Rabaptin-5 localisation to early endosomes is also conserved in *Drosophila* as we show that depletion of Rabex-5 levels using RNAi causes a reduction in Rbpn-5 puncta. This is surprising as not only does Rbpn-5 contain a Rab5 binding domain, but also a FYVE domain (unlike its mammalian homologue), which would be expected to promote its recruitment to early endosomes. It is possible that the FYVE domain is non-functional. Alternatively, its ability to bind PI3P may be somehow inhibited in the absence of Rabex-5, or the protein may be destabilised. Further work would be required to distinguish between these possibilities.

We found that over-expressing a mutant form of Rabex-5 lacking GEF activity (Yan et al., 2010) prevents both Rbpn-5 and Rab5 from accumulating in apical puncta. We surmise that the construct is acting in a dominant-negative manner to suppress Rbpn-5 recruitment and/or Rab5 activation via endogenous Rabex-5. In mammalian cells, localisation of all three proteins appears to be highly interdependent, suggesting that it is likely that Rabex-5 recruitment to early endosomes is also compromised in the absence of Rbpn-5 or Rab5. However, in the absence of reagents for visualising endogenous Rabex-5, this remains speculative.

### *Rbpn-5* and *Rabex-5* neoplastic discs do not show disrupted apico-basal polarity

The mechanistic basis of neoplastic tumour formation is complex and much remains unclear. *Drosophila* models, in particular the classic baso-lateral polarity complex mutants *scrib*, *dlg* and *lgl*, which were discovered many years ago and which, unlike most of the endocytic mutants, are zygotically viable until late larval stages, have been extremely useful in describing the process of tumorigenesis (Bilder et al., 2000; Bilder and Perrimon, 2000; Bryant and Schubiger, 1971; Gateff, 1978; Gateff and Schneiderman, 1967; reviewed in Enomoto and Igaki, 2011). There are several different pathways and mechanisms that have been investigated. Given that Scrib, Dlg and Lgl regulate apico-basal polarity by inhibiting the apical Crb and Par-3/Bazooka (Baz) complexes (Betschinger et al., 2003; Bilder et al., 2003; Tanentzapf and Tepass, 2003), an expansion of the apical domain was proposed as one possible mechanism for tumourigenesis. This hypothesis was reinforced by the finding that over-expressing either Crb or atypical Protein kinase C (aPKC) is sufficient to promote tumourous discs, and that in the endocytic mutants, Crb accumulates massively and apico-basal polarity is disrupted (Bilder and Perrimon, 2000; Leong et al., 2009; Lu and Bilder, 2005; Moberg et al., 2005; Vaccari and Bilder, 2005). Crb accumulation is thought to promote the growth aspect of the neoplastic tumour phenotype through misregulation of the Hpo/Wts pathway (Chen et al., 2010; Grzeschik et al., 2010; Ling et al., 2010; Robinson et al., 2010; Robinson and Moberg, 2011). However, there is some evidence that Crb is not always strongly misregulated in neoplastic tumours, and it has been speculated that in *scrib*, *dlg* and *lgl* mutants it may be the concurrent disruption of endocytosis, rather than the polarity defect, which is the primary cause of their neoplastic phenotypes (Leong et al., 2009; Robinson and Moberg, 2011).

Our results show that neither *Rabex-5* nor *Rbpn-5* exhibit disruption in apico-basal polarity and there is no accumulation of Crb (Figs. 1F-J, 5B-D). We suggest that these mutants are likely to represent a milder phenotype than the other endocytic mutants studied so far, possibly due to enhanced perdurance of maternal protein which allows the survival of zygotic mutants to early pupal stages. In any case, the lack of an apico-basal polarity defect in *Rbpn-5* and *Rabex-5* mutants indicates that loss of apico-basal polarity is not strictly required for neoplasia.

### Mechanisms of neoplastic tumour formation in *Rbpn-5* and *Rabex-5* mutants

Other pathways that have been found to be activated in *Drosophila* neoplastic tumours include N, JAK/STAT and JNK. Due to the differential ability of N to signal in different endocytic compartments, the pathway is upregulated in ESCRT mutant tumours, but not in early endocytic mutants, despite their accumulation of N protein on the cell surface (Herz et al., 2006; Herz et. al., 2009; Lu and Bilder, 2005; Moberg et al., 2005; Morrison et al., 2008; Thompson et al., 2005; Vaccari and Bilder, 2005; Vaccari et al., 2008). As with Crb, we do not observe accumulation of N in *Rbpn-5* or *Rabex-5* mutant discs (Figs. 6A-B, and S2C), suggesting that a certain level of endocytosis is still occurring, and unsurprisingly we also find that the N pathway is not activated.

In ESCRT mutant clones, activation of the N pathway leads to transcription of the JAK/STAT ligand Upd, which acts non-autonomously on wild-type tissue to induce JAK/STAT signalling and promote neoplasia (Herz et al., 2006; Moberg et al., 2005; Vaccari and Bilder, 2005). The massive upregulation of JAK/STAT signalling that we observe must be activated via an alternate mechanism, as it is both cell autonomous and N-independent. Although autonomous JAK/STAT signalling in neoplastic discs has been documented and shown to contribute to excessive cell size and proliferation (Gilbert et al., 2009; Woodfield et al., 2013), it is unclear how it is activated in endocytic mutants. One explanation that we were unable to test, is that the endocytic block could directly disrupt the trafficking of the JAK/STAT receptor Domeless (Dome), as Dome localisation has been shown to be altered in ESCRT mutant neoplastic tumourous discs (Gilbert et al., 2009), and signalling ability is known to be influenced by the intracellular compartment in which the ligand-receptor complex finds itself (Devergne et al., 2007; Vidal et al., 2010). We show that a hypomorphic *stat92E* mutation is able to rescue many of the neoplastic defects, including over-proliferation, inability to differentiate and overall disc shape and structure, suggesting that ectopic activation of JAK/STAT signalling in endocytic mutants is indeed causative for these aspects of tumourigenesis.

The upregulation of the JNK pathway that we observe in the *Rbpn-5* and *Rabex-5* mutants is likely to also contribute to the over-proliferation phenotype. The JNK pathway is unusual in having both pro-proliferation and pro-apoptotic roles, and was initially thought to be activated only where wild-type tissue abuts clones mutant for neoplastic tumour suppressor genes, promoting apoptosis within mutant cells and their elimination from the tissue (Brumby and Richardson, 2003; Igaki et al., 2006, 2009; Ohsawa et al., 2011; Uhlirova et al., 2005). However, more recent work has shown that JNK signalling is also activated in tissues wholly mutant for endocytic neoplastic tumour suppressor genes where it promotes cell proliferation (Woodfield et al., 2013).

It is thought that JNK pathway upregulation in neoplastic tumours is likely to act through the Eiger/TNF ligand receptor complex (Igaki et al., 2002, 2009; Moreno et al., 2002). Eiger and activated JNK have been shown to accumulate in early endosomes in *scrib* mutant clones to promote apoptosis, and there are several other pieces of evidence that implicate the endocytic pathway in Eiger regulation (Igaki et al., 2009). Interestingly, the switch from JNK-mediated apoptosis to proliferation can be mediated by co-expression of the *Ras* oncogene. For example, expressing Ras in *scrib* mutant clones produces a highly invasive neoplastic phenotype such that clones that would usually be eliminated instead invade neighbouring wild-type tissue (Brumby and Richardson, 2003; Doggett et al., 2011; Igaki et al., 2006; Pagliarini and Xu, 2003; Uhlirova et al., 2005; Wu et al., 2010). Given that Ras is ubiquitinated by Rabex-5, and the Ras pathway has been shown to be upregulated in *Rabex-5* mutant larvae (Xu et al., 2010; Yan et al., 2010), it is intriguing to speculate whether this may be contributing in some way to the neoplastic phenotype seen in mutant larval discs, not only in the *Rabex-5* background but perhaps more generally in other endocytic neoplastic tumour suppressor mutants. Indeed, as Rabex-5-mediated ubiquitination targets cargo to early endosomes for degradation (Aikawa, 2012; Aikawa et al., 2012; Mattera and Bonifacino, 2008; Mattera et al., 2006), it seems highly likely that Ras signalling is not only regulated by ubiquitination but also by endosomal dynamics. Further work will be needed to investigate a potential role for Ras in endocytic neoplastic tumours, to determine the mechanisms of JNK and JAK/STAT activation and to elucidate how differential activity of these pathways is controlled in clones *versus* whole mutant tissues.

## Footnotes

Appendix A Supplementary data associated with this article can be found in the online version at doi:10.1016/j.ydbio.2013.09.029.
